# Draft genome sequence of the deep-subsurface *Ciceribacter* sp. strain T2.26MG-112.2, a second Rhizobiaceae isolated from the Iberian Pyrite Belt at 492.6 mbs

**DOI:** 10.1128/mra.00502-23

**Published:** 2024-04-02

**Authors:** José M. Martínez, R. García, T. Leandro, R. Amils

**Affiliations:** 1 Scientific Program Interactions with the Environment, Molecular Ecology of Extreme Environments, Centro de Biología Molecular Severo Ochoa (CBMSO, CSIC-UAM), Madrid, Spain; 2 Grupo de investigación de alto rendimiento en Ingeniería Química y Ambiental, Escuela Superior de Ciencias Experimentales y Tecnología, Universidad Rey Juan Carlos (URJC), Móstoles, Spain; 3 Institute for Bioengineering and Bioscience, Instituto Superior Técnico, Lisbon, Portugal; 4 Planetology and Habitability Department, Centro de Astrobiología (CAB, INTA-CSIC), Torrejón de Ardoz, Spain; SUNY College of Environmental Science and Forestry, Syracuse, New York, USA

**Keywords:** *Ciceribacter*, Rhizobiaceae, deep subsurface

## Abstract

T2.26MG-112.2 is a *Ciceribacter* strain that has been isolated from the deep subsurface of the Iberian Pyrite Belt. We report its draft genome consisting of a chromosome of ≈4.9 Mb and a plasmid of 357 kb. The annotation reveals 4,824 coding sequences, 48 tRNA genes, and 1 rRNA operon.

## ANNOUNCEMENT

The family Rhizobiaceae includes 16 genera, of which *Rhizobium* is one of the main ones. Most *Rhizobium* species can establish symbiotic relationships with legumes, although free-living species were also described. Currently, the genus *Rhizobium* has been revised and reordered in new genera. Particularly, *Rhizobium selenitireducens* and *Rhizobium naphthalenivorans* have been reclassified in the genus *Ciceribacter* (1). The facultative aerobe *Ciceribacter* sp. strain T2.26MG-112.2 was isolated from a strict anaerobic methanogenic enrichment culture derived from a 492.6-m deep core sample obtained from a drilling project, IPBSL (2). Drilling was performed as described by Amils et al. (2). The isolation and growth of *Ciceribacter* sp. strain T2.26MG-112.2 were done using an enrichment medium for methanogenic microorganisms with an atmosphere of H_2_:CO_2_ (80:20, vol/vol) described in reference 3.

Genomic DNA of the strain T2.26MG-112.2 was extracted using the cetyltrimethylammonium bromide method (4). The 16S rRNA gene was amplified with a 27F/1492R pair of primers and compared with the GenBank database of NCBI, via BLAST, as described in references 5, 6. The closest sequence was found to be *Ciceribacter naphtalenivorans* TSY03b^(T)^ (98.88%). Genomic DNA was sequenced by Illumina MiSeq platform, with 32.3× mean coverage whose results were 380,110 paired-end reads with a length of 36–251 bp. A Nextera XT kit (Illumina) was used to prepare the genomic libraries following the manufacturer’s protocol. The trimming of the reads was done with Trimmomatic software by the MicrobesNG facility. The quality of reads was examined with the FastQC v0.12.0 software. *De novo* assembly was performed by SPAdes 3.15.3 (7), and the assembly of extra-chromosomal genetic elements was done using PlasmidSPAdes (8). These plasmid contigs were aligned against the chromosomal assembly with MAUVE Aligner (9) to separate chromosomal and plasmid contigs. Contigs were ordered and new scaffolds were created via SSPACE (10) giving a chromosome of 4,974,762 bp in 80 scaffolds with an *N*_50_ of 212 kb, and a GC content of 62.95%. Likewise, a plasmid with a size of 357,041 bp in six scaffolds with an *N*_50_ of 129 kb has been obtained whose length is comparable with the length of plasmids reported from other members of the family Rhizobiaceae (150–400 kb) (11). Default parameters were used for all software unless otherwise specified.

Gene prediction and annotation were achieved using PROKKA software (12) and the RAST platform (13) taking the genome of *Rhizobium leguminosarum* bv viciae 3841 as a reference. A total of 4,824 coding DNA sequences, 459 signal peptide genes, 48 tRNA genes, and 1 rRNA operons were identified. Complete genes encoding for denitrification, dissimilatory nitrate reduction to ammonium, and a series of highly conserved reductase enzymes, which are part of terminal electron acceptors in anaerobic respiration, have been identified on the chromosome as well as *Ciceribacter* T2-30D-1.1 (5), another *Cicerobacter* isolated at other depths (2).

As in the case of *Ciceribacter* sp. T2.30D-1.1 (5), *vir* genes associated with virulence in plants were found in the plasmid of T2.26MG-112.2. In addition, genes involved in plasmid replication (*rep*A, *rep*B, and *rep*C) and conjugation were also identified.

## Data Availability

Reads have been deposited at DDBJ/ENA/GenBank under the accession number ERR2572853, and the complete genome sequences and annotations have been deposited under the accession numbers UEYQ01000000 for this genome and UFQV01000000 for this plasmid. This version described in this paper is the first version.
